# Rectus Abdominis Endometriosis Following Cesarean Section: A Case Report

**DOI:** 10.7759/cureus.55462

**Published:** 2024-03-03

**Authors:** Areti Kalfoutzou, Asimina Restemi, Adam Mylonakis, Konstantinos Papadimitropoulos, Dimitrios Matsaridis, Andria Peraki, Margaritis Tsantopoulos, Nikolaos Chaleplidis

**Affiliations:** 1 Department of Medical Oncology, 251 Air Force General Hospital, Athens, GRC; 2 Department of Pathology, 251 Air Force General Hospital, Athens, GRC; 3 Department of Surgery, Laikon General Hospital, National and Kapodistrian University of Athens, Athens, GRC; 4 Department of Surgery, 251 Air Force General Hospital, Athens, GRC; 5 Department of Radiology, 251 Air Force General Hospital, Athens, GRC; 6 Department of Gynecology, Elena Venizelou General Maternal Hospital, Athens, GRC

**Keywords:** cesarean section, surgical margins, surgical excision, caesarian section, rectus abdominis muscle, endometriosis

## Abstract

Endometriosis involves the growth of endometrial-like tissue outside the uterine cavity, with its manifestation in the rectus abdominis muscle being exceptionally rare and primarily observed in women with a history of abdominal surgeries. In this report, we present the case of a 42-year-old female with a medical history of two cesarean sections who presented with cyclical abdominal pain and a palpable mass in the right lower quadrant. An MRI scan of the pelvis revealed a lesion on the right lower quadrant of the abdominal wall, proximate to the previous Pfannenstiel incision. A percutaneous US-guided biopsy of the abdominal lesion was performed, and histopathology demonstrated the presence of endometrial glands and stroma, confirming the diagnosis of rectus abdominis endometriosis. She was submitted to a local wide excision with adequate margins of normal surrounding tissue and has remained free of recurrence for two years.

## Introduction

Endometriosis, characterized by the presence of endometrial tissue outside the uterus, is a complex gynecological condition affecting approximately 5%-10% of women globally [1,2]. Ectopic endometrial tissue, while most commonly found within the pelvic cavity (on the ovaries, fallopian tubes, and peritoneum) can, in rarer instances, be present in extrapelvic locations such as the abdominal wall, lung, or even the brain. [1,3]. Notably, the prevalence of abdominal wall endometriosis is reported to be between 0.03% and 1% among women who have undergone cesarean sections or other types of abdominal surgery [4]. Despite its rarity, the impact of abdominal wall endometriosis on patients can be profound, often resulting in significant pain and discomfort, which may be cyclical in nature, coinciding with the menstrual cycle. The diagnosis of rectus abdominis endometriosis poses significant challenges, as its symptoms can overlap with those of other abdominal pathologies, necessitating a thorough differential diagnosis process and often requiring a multidisciplinary approach. This report presents the case of a 42-year-old woman presenting with cyclical abdominal pain and a palpable abdominal wall mass who was diagnosed with rectus abdominis endometriosis. The case aims to highlight the distinct clinicopathological characteristics and to increase clinical awareness and diagnostic suspicion of endometriosis in this uncommon location, particularly in women with a history of abdominal surgeries.

## Case presentation

A 42-year-old woman was referred to our department due to a history of abdominal pain that intensified during menstruation over the past four years. Her medical history included two cesarean sections, performed 12 and 10 years ago, and the surgical excision of an in situ melanoma in the genital region seven years ago. She had no reported history of pelvic endometriosis. A physical examination of the abdomen revealed a firm, painless, palpable mass in the lower right quadrant. Laboratory values, including tumor marker cancer antigen 125 (CA-125), were within normal limits (17.4, normal range: 0-35 U/mL). 

**Figure 1:**
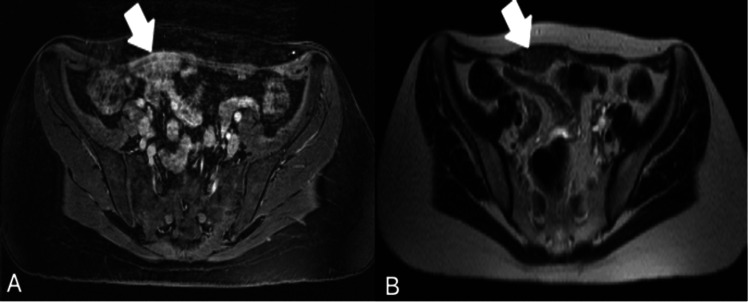
An MRI scan of the abdomen with intravenous contrast Panel A: T1 post-contrast fat-saturated weighted image depicts a lesion with avid enhancement in the right rectus abdominis muscle (white arrow). Panel B: A T2-weighted image reveals a lesion with higher signal intensity in the right rectus abdominis muscle compared to the left (white arrow).

A transvaginal ultrasound showed no abnormal findings. However, an abdominal ultrasound identified a focal hypoechoic lesion within the rectus abdominis muscle. Further evaluation by MRI scan of the pelvis revealed a lesion measuring 20 mm in its largest dimension, located on the right lateral aspect of the lower-anterior abdominal wall, in proximity to the Pfannenstiel incision. On T1-weighted fat-saturated images, the lesion exhibited avid contrast enhancement, whereas on T2-weighted images, its signal was slightly hyperintense compared to the left rectus abdominis muscle (Figure 1). Notably, the posterior edge of the lesion was closely adjacent to loops of the small intestine without intruding upon them.

**Figure 2:**
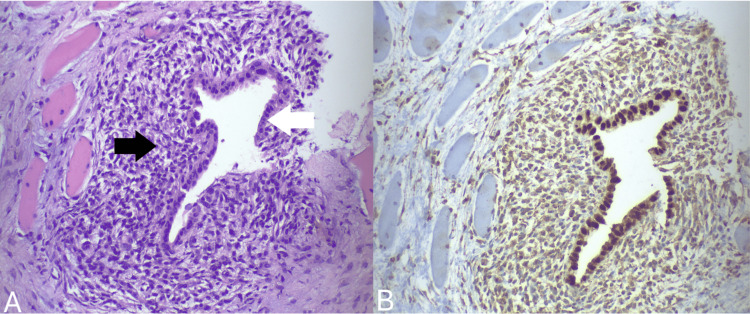
Panel A: Histopathological examination (hematoxylin-eosin stain, 20x magnification) exhibits endometrial glands (white arrow) and stroma (black arrow) embedded within muscle fibers (visible on the left side of the image). Panel B: In the immunohistochemical analysis (20x magnification), positive PAX8 staining is observed in both the glandular component and the surrounding stroma. PAX8: paired-box gene 8

**Figure 3:**
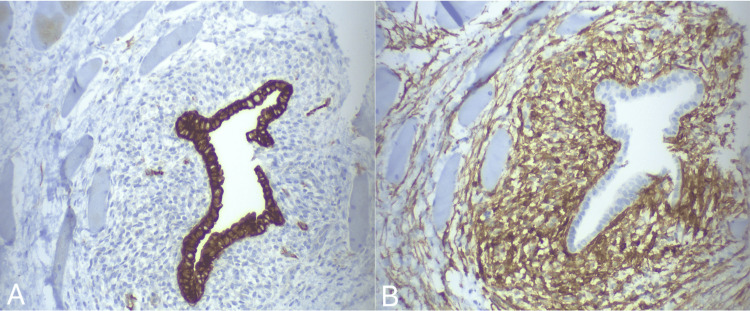
Immunohistochemical staining, 20x magnification Panel A: The glandular component exhibits positive staining for CK7. Panel B: The stromal component shows positive staining for CD10. CK7: cytokeratin 7

A percutaneous US-guided biopsy of the palpable lesion was performed. Histopathological examination revealed the presence of endometrial-type glands and stroma embedded within the muscular tissue (Figure 2). Additionally, foci of hemosiderin-laden foci, fibrosis, and atrophy were observed in the adjacent muscular tissue. Immunohistochemical staining showed that the glandular cells were positive for cytokeratin 7 (CK7) and paired-box gene 8 (PAX8), while the stromal cells exhibited positive staining for CD10 (Figure 3). Collectively, these findings were suggestive of ectopic endometrial tissue strictly confined within the rectus abdominis muscle without extension to adjacent tissues.

Subsequently, the patient underwent local excision of the lesion en bloc with part of the surrounding rectus abdominis muscle without entering the peritoneal cavity. Macroscopic examination of the surgical specimen revealed a whitish lesion measuring 25 mm in size. The lesion was removed with a minimum margin of 12 mm of surrounding normal tissue on all sides. The lesion demonstrated characteristic features of endometriosis, including endometrial glands and stroma, accompanied by both recent and old hemorrhagic activity. Hemosiderin-laden macrophages, as well as focal atrophy, were observed in the surrounding muscle tissue. Immunohistochemical analysis showed positive staining for CK7 in the glandular cells and positive staining for CD10 in the stromal cells. Both glandular and stromal cells expressed PAX8, estrogen, and progesterone receptors, confirming the diagnosis of rectus abdominis endometriosis. 

The postoperative course was uneventful, and the patient was discharged the following day. She has been under consistent gynecological follow-up for two years since the surgery, including monitoring with CA-125 measurements, abdominal MRI scans, and gynecological ultrasounds, with no evidence of disease recurrence. The patient is scheduled for biannual follow-up visits for a total of five years.

## Discussion

Endometriosis is defined by the presence of functional ectopic endometrial tissue outside the uterine cavity [5,6]. This condition primarily affects women of childbearing age, with a peak incidence around the age of 35, and is a major cause of infertility among women in this age group [5]. Endometriosis can manifest within the pelvic cavity, affecting the ovaries, fallopian tubes, Douglas pouch, cervix, vagina, bladder, rectum, and peritoneal surface, or extend to extrapelvic organs such as the skin, kidneys, lungs, extremities, liver, stomach, or brain [2,4,6-8]. An exceedingly rare form is rectus abdominis endometriosis, where the endometrial tissue lies within the rectus abdominis muscle [3,4]. This particular type of endometriosis predominantly affects women who have undergone prior abdominal surgical procedures, such as cesarean sections, with the onset usually ranging from three months to 10 years after the surgery [4,8,9]. The prevalence of rectus abdominis endometriosis, especially following a cesarean section, is reported to range from 0.03% to 0.45% [8]. Approximately 25% of these cases have a history of pelvic endometriosis [6,10,11]. Our patient presented symptoms six years after a cesarean section, and there was no history of pelvic endometriosis.

Various theories have been suggested to explain the etiology of endometriosis. The transplantation theory, widely recognized, hypothesizes that endometriosis develops from the retrograde transport of viable endometrial tissue during menstruation [6,7,9,12,13]. The coelomic metaplasia theory proposes that endometriosis stems from the transformation of peritoneal cells [6,9,12,13]. The induction theory, alternatively, suggests that the spread of shed endometrial tissue leads to endometriosis [7,10,12]. Moreover, the embryonic rest theory proposes that endometriosis is triggered by the activation of Müllerian origin cells [10]. Notably, abdominal wall endometriosis has been linked to iatrogenic dissemination following surgical procedures in the abdomen [7]. Furthermore, the potential for lymphatic and vascular spread has been considered in the pathogenesis of extrapelvic endometriosis [7,10,12-14]. 

The clinical presentation of rectus abdominis endometriosis varies widely, ranging from asymptomatic to severe, incapacitating acute abdominal pain [11,15]. The most common symptoms include abdominal pain that intensifies during menstruation, dysmenorrhea, irregular menses, dyspareunia, or infertility [2,8]. Non-cyclical abdominal pain can also be a symptom of this condition in one-third of patients [14]. A palpable nodule may be detected during a clinical examination, typically located adjacent to a surgical scar [16]. In rare instances, a diagnosis is made incidentally during a surgical procedure performed for a different medical issue [17]. 

Laboratory tests typically do not yield diagnostic results, although CA-125 levels are frequently found to be mildly elevated [2,3]. Ongoing studies are evaluating the potential of markers like C-reactive protein (CRP), anti-Müllerian hormone (AMH), follistatin, CA 19-9, CA 15-3, and vascular endothelial growth factor (VEGF), among others, as indicators for the detection of endometriosis; yet, to date, none have shown considerable specificity [3,11]. In our case, all laboratory tests, including CA-125, were within normal ranges.

Imaging modalities, including ultrasonography, CT scans, and MRI scans, can aid in distinguishing rectus abdominis endometriosis from adjacent tissues [15]. Common ultrasonographic findings include well-defined, hypoechoic masses that may be solid, cystic, or have mixed components and frequently exhibit high vascularity on Doppler imaging [2,6,9,16]. On a CT scan, the lesions generally appear isointense to muscle tissue and exhibit heterogeneous, significant contrast enhancement [16]. An MRI scan of the abdomen and pelvis is the preferred imaging modality, often showing cystic structures that display high signal intensity on T1-weighted images and variable intensities on T2-weighted images, depending on the amount of glandular tissue and the age of hemorrhagic products (9,15,16). However, it is important to note that there are no imaging findings pathognomonic for diagnosing rectus abdominis endometriosis [1,2,6,16].

Differential diagnosis includes a variety of benign and malignant lesions of the abdominal wall, such as suture granuloma, lymphadenopathy, abscess, inguinal or incisional hernia, lipoma, hematoma, subcutaneous and sebaceous cysts, primary or metastatic cancer, lymphoma, sarcoma, and desmoid tumor [2,10-12,17-19]. 

A definitive diagnosis is established through histopathological analysis of tissue samples, typically acquired via a US-guided biopsy [6,16,18]. In certain instances, the diagnosis is established postoperatively following an excisional biopsy [18]. Histopathological examination typically shows the presence of endometrial glands and stroma embedded in the skeletal muscle fibers of the rectus abdominis muscle [10,14]. The glands are encased by columnar epithelial cells, while the stroma consists of small, spindle-shaped cells with minimal cytoplasm [9]. Occasional foci of hemosiderin-laden macrophages can also be observed within the striated muscle [20]. Immunohistochemistry typically indicates positive staining for estrogen and progesterone receptors in both stromal and glandular cells, as well as positive staining for PAX8 and CK7 in glandular cells and positive CD10 staining in endometrial stroma [6,21].

The primary treatment for this condition is wide local excision, which necessitates the removal of at least 10 mm of normal tissue surrounding the lesion to reduce the risk of recurrence [2,7,9,17]. In rare instances, asymptomatic cases may be monitored without immediate intervention [15]. Historically, pharmacological treatments such as oral contraceptives, gonadotropin-releasing hormone (GnRH) analogs, progesterone, or danazol have been used with limited effectiveness as primary treatments [3,7,9,16]. However, these agents may serve as an alternative for patients who cannot undergo surgery or as adjuvant therapy to mitigate the risk of recurrence [7,9,16]. There has been a report of successful treatment of rectus abdominis endometriosis using sclerotherapy with ultrasound-guided ethanol injection [18]. Additionally, high-intensity focused ultrasound (HIFU) ablation has been experimented with and has shown promising results [22]. In our case, surgical intervention was decided due to the patient’s escalating abdominal pain, which led to the complete resolution of symptoms. 

The prognosis is typically favorable, with a recurrence rate of up to 4.3% [9,11]. Instances of malignant transformation have been documented, occurring in roughly 0.3% to 1% of cases [9,11]. Regular gynecological follow-up is crucial to detect any potential recurrence of endometriosis in the local area. This involves clinical assessment, CA-125 measurements, as well as consistent gynecological ultrasounds and abdominal imaging [10,11]. 

To the best of our knowledge, this is the 25th case of endometriosis strictly confined within the rectus abdominis muscle reported in the literature since 1984 [3,6,7,23-26]. The patient's history of cesarean sections, the cyclical nature of her pain in sync with menstrual cycles, and the detection of a palpable nodule during clinical examination are hallmark features of rectus abdominis endometriosis. This case underscores the importance of considering rectus abdominis endometriosis in differential diagnosis, particularly in patients with similar clinical profiles, to ensure timely and appropriate management.

## Conclusions

The diagnosis of rectus abdominis endometriosis requires a high index of suspicion, especially in women with a history of abdominal surgeries presenting with cyclical abdominal pain. Surgical excision, ensuring wide margins of normal tissue, emerges as the cornerstone of treatment, offering the most definitive means of symptom relief and minimizing the risk of recurrence. Ongoing and thorough follow-up of patients is imperative in ensuring early recurrence detection and in maintaining and potentially enhancing the patient's overall quality of life.
